# A Case of Leukocytoclastic Vasculitis and Associated Conjunctivitis Following MMR Vaccine Administration

**DOI:** 10.1155/2023/9001287

**Published:** 2023-02-06

**Authors:** Hana I. Nazir, Aubrey A. Hess, Abha Soni, Kathryn A. Potter

**Affiliations:** ^1^Medical College of Georgia, Augusta, GA, USA; ^2^Contra Costa Pathology Associates, Pleasant Hill, CA, USA

## Abstract

**Introduction:**

Leukocytoclastic vasculitis (LCV) is a small-vessel vasculitis characterized by immune complex deposition in the walls of dermal capillaries and venules. With the COVID-19 pandemic, more adults are receiving the MMR vaccine, as it may enhance innate immune responses against COVID-19 infection. Here, we report a case of LCV and associated conjunctivitis arising in a patient secondary to immunization with the MMR vaccine.

**Methods and Results:**

A 78-year-old man on lenalidomide therapy for multiple myeloma presented to an outpatient dermatology clinic with a two-day history of a painful rash consisting of scattered pink dermal papules on bilateral dorsal and palmar hands, as well as bilateral conjunctival erythema. Histopathological findings—which revealed an inflammatory infiltrate with papillary dermal edema, as well as nuclear dust within small blood vessel walls with extravasation of red blood cells—were most consistent with LCV. It then became known that the patient had received an MMR vaccine two weeks prior to the onset of the rash. The rash was resolved with the use of topical clobetasol ointment, and the patient's eyes were cleared as well.

**Conclusions:**

This is an interesting presentation of MMR vaccine-related LCV occurring only on the upper extremities with associated conjunctivitis. Had the patient's oncologist not known about the recent vaccination, it is likely that the treatment of his multiple myeloma would have been postponed or altered, as lenalidomide can also cause LCV.

## 1. Introduction

Leukocytoclastic vasculitis (LCV) is a small-vessel vasculitis characterized by immune complex deposition in the walls of dermal capillaries and venules. While the majority of cases of LCV are idiopathic, the condition is often associated with infections and newly administered medications [1]. Here, we report a case of LCV and associated conjunctivitis arising in a patient secondary to immunization with the measles, mumps, and rubella (MMR) vaccine.

## 2. Case Report

A 78-year-old man with a one-year history of lenalidomide therapy for multiple myeloma presented to an outpatient dermatology clinic with a two-day history of a painful rash on his hands, as well as red, painful eyes. He denied any recent upper respiratory infection or having started any new medications.

**Figure 1:**
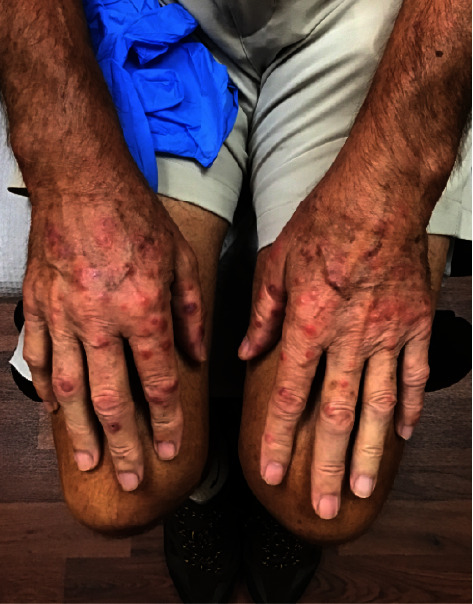
Patient presentation at rash onset.

At the clinic visit, physical examination was notable for scattered pink dermal papules with a dusky red center bilateral dorsal and palmar hands, as well as bilateral conjunctival erythema (Figure 1). Differential diagnoses included erythema multiforme, acute febrile neutrophilic dermatosis, erythema elevatum diutinum, and multicentric reticulohistiocytosis. A punch biopsy was taken from the left index finger.

**Figure 2:**
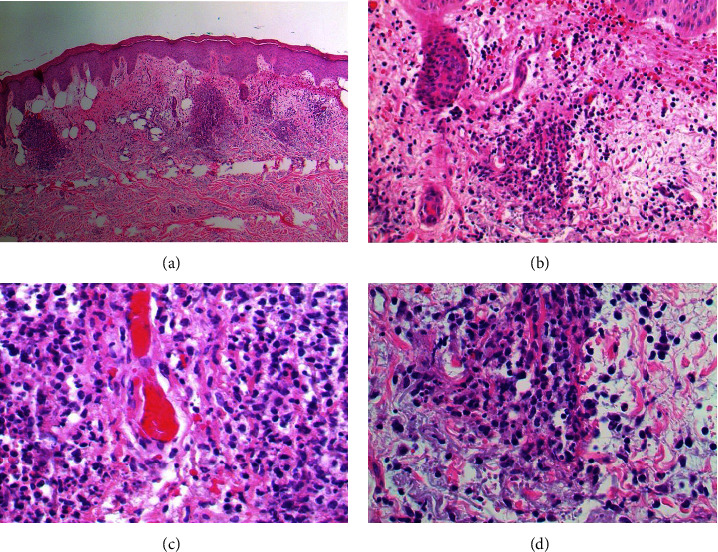
Histology on low (hematoxylin-eosin stain; original magnifications: (a) x 20) and high (hematoxylin-eosin stain; original magnifications: (b) x 200) power show a moderately dense perivascular and interstitial inflammatory infiltrate composed of lymphocytes, histiocytes, and numerous neutrophils with surrounding papillary dermal edema. (c)/(d). High power views demonstrate scattered nuclear dust (karyorrhexis) and fibrin within the walls of the small blood vessels with extravasation of red blood cells in the surrounding stroma (hematoxylin-eosin stain; original magnifications: (c) x 400; (d) x 400).

**Figure 3:**
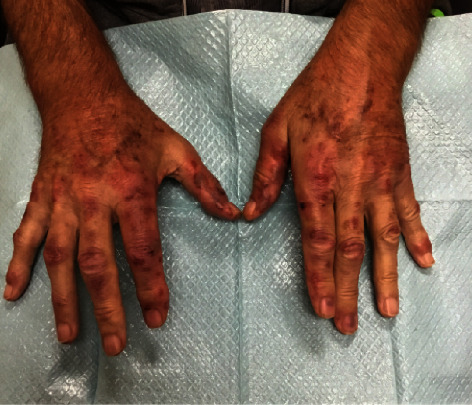
Rash at one week follow-up.

Histological examination revealed a dense perivascular and interstitial inflammatory infiltrate composed of lymphocytes, histiocytes, and numerous neutrophils with surrounding papillary dermal edema. High-power views demonstrated scattered nuclear dust and fibrin within the walls of the small blood vessels with extravasation of red blood cells in the surrounding stroma (Figure 2). The patient's histopathological findings were most consistent with leukocytoclastic vasculitis (LCV). Upon discussion with the patient's oncologist, it became known that the patient had received an MMR vaccine two weeks prior to the rash. The patient did not have a history of COVID-19 vaccination. The patient followed up one week later with a more typical appearing rash for LCV (Figure 3). The rash was resolved with the use of topical clobetasol ointment and his eyes were also cleared. The patient has continued to receive lenalidomide without any recurrence of the rash.

## 3. Discussion

Leukocytoclastic vasculitis is a small-vessel vasculitis characterized by immune complex deposition in the walls of dermal capillaries and venules. LCV commonly presents as erythematous macules with palpable purpura bilaterally, often in the lower extremities. LCV has previously been associated with vaccines, including vaccinations to influenza and herpes zoster [2–4].

MMR vaccine-induced LCV in adults, accompanied by conjunctival injection has rarely been described. Typically, the MMR vaccine is indicated for children and adults born before 1957 [5]. With the recent COVID-19 pandemic, more adults are receiving the MMR vaccine as it may induce innate immune responses that provide protection against COVID-19 infection via decreased production of proinflammatory cytokines [6].

While the MMR vaccine rarely results in serious adverse effects, it has been associated with fever, transient rashes, transient lymphadenopathy, and parotitis in certain cases [5]. Additionally, subtypes of leukocytoclastic vasculitis in children, such as Henoch-Schonlein purpura and acute hemorrhagic edema of infancy have been reported following MMR vaccine [7, 8]. There is one case report of a patient who developed LCV on her upper extremities and uveitis associated with the MMR vaccination [9].

Given that lenalidomide can also cause LCV, the history of recent vaccination was important for this patient to be treated appropriately [10]. Had his oncologist not known about the recent MMR vaccination, it is likely that the treatment of his multiple myeloma would have been postponed or altered. In this case, it is unlikely that lenalidomide triggered the patient's vasculitis given that the patient has continued to receive lenalidomide without recurrence of the rash. Moreover, the patient had a longstanding prior history of lenalidomide therapy without any occurrence of LCV.

This is an interesting presentation of LCV occurring only on the upper extremities with associated conjunctivitis. As more adults are receiving the vaccine during the COVID-19 pandemic, it is important for clinicians to be aware of potential side effects and treat them promptly and properly.

## Additional Points

As more adults are receiving MMR vaccines during the COVID-19 pandemic, it is important for clinicians to be aware of leukocytoclastic vasculitis as a potential adverse effect in order to treat their patients promptly and properly.
